# Fellowship Training After Four-year Emergency Medicine Residency

**DOI:** 10.5811/westjem.62105

**Published:** 2026-05-19

**Authors:** Michael R. Ehmann, Eili Y. Klein, Gabor D. Kelen

**Affiliations:** Johns Hopkins University School of Medicine, Department of Emergency Medicine, Baltimore, Maryland

To the Editor:

We read with interest Dr. Hamilton et al’s letter of concern regarding the potential implications of requiring four years of training for all emergency medicine residency programs.^1^ The authors raise several considerations that warrant discussion. Principally, we wish to clarify their interpretation of the relationship between residency length of training and the likelihood of pursuing fellowship.

The authors cite our 2021 paper, “Emergency Medicine Career Outcomes and Scholarly Pursuits: The Impact of Transitioning from a Three-year to a Four-year Niche-based Residency Curriculum,” to suggest that requiring a standard four-year training format may lead to a reduction in the number of graduates pursuing fellowship training.^2^ This assertion is not supported by our paper. Our multivariable statistical analysis found that four-year residency graduates were no less likely to pursue fellowship opportunities than when our program was a three-year format (odds ratio 0.58, 95% CI, 0.18–1.87; Table 2).^2^

Our manuscript is the sole source of evidence provided for the authors’ opinion regarding the effect of residency length on fellowship applications. Therefore, we believe it would have been more appropriate for them to temper their assertion in the absence of any other corroborating support since there is evidence going back 20 years that graduates of four-year EM programs were *more* likely to pursue fellowship training.^3^ More recently, a 2024 study demonstrated that residents’ career decisions, including whether to pursue a fellowship, are influenced by multiple factors—including mentorship, perceived career needs, and subspecialty exposure during residency—all of which may be positively affected by a fourth year of residency training.^4^

Further, since our original paper was published, five classes have graduated from our four-year residency. Of these 59 recent graduates, 18 (30.5%) have pursued fellowship training. When these alumni are included in an updated analysis following the same methods described in our original manuscript, the observed absolute difference in the number of residents pursuing a fellowship is inverted from 5.4% in favor of a three-year format to 5.6% in favor of a four-year format. The odds ratio of pursuing a fellowship remains statistically non-discernible (odds ratio 1.29, 95% CI, 0.64–2.58). While this reflects the experience of a single program and our observations could be affected by self-selection bias, this updated analysis affirms our 2021 observation that four-year program graduates are not less likely to pursue fellowship than their three-year colleagues, and in more recent years, may be even more likely to do so.^2^

We appreciate Dr. Hamilton et al’s contributions to this important discussion on behalf of the Association of Academic Chairs of Emergency Medicine but respectfully disagree with their interpretation of our work and with their conclusions about the potential implications of the ACGME’s proposed program requirements.
